# Supplementary material for: Probing the chemical stability between current collectors and argyrodite Li6PS5Cl sulfide electrolyte
Source: Commun Chem. 2025 Jul 24;8:212. doi: 10.1038/s42004-025-01609-9 (PMC12289912; doi:10.1038/s42004-025-01609-9)
Supplement: Supplementary file 2 — Supplementary Information [file 42004_2025_1609_MOESM2_ESM.pdf]

Supplementary Information

Methods

All the electrochemical tests were carried out in CR2032 button cells, which were provided by the supplier (for example, X2lab and Hohsen Corp. (Table S1, Supplementary Information)). The thickness of the sample and the spring used, which is undulating, therefore determine the pressure applied during cycling. Considering a total sample thickness of 1.9 mm (without the spring), the pressure inside a CR2032 setup was determined to be 0.2 MPa [1,2].

Materials: Lithium metal foils (MTI, Li Chips, diameter 15.6 mm, thickness 0.25 mm) and Li on Cu foils were purchased from China Energy Lithium Co. (width 80 mm, thickness of Li 0.04 mm). Current collectors (CC) made of copper foil (Cu, diameter 15 mm, thickness 0.15 mm), steel washers (SS316, diameter 15.5 mm, thickness 0.5 mm), nickel foil (Ni, diameter 15 mm, thickness 0.15 mm), aluminium foil (Al, diameter 15 mm, thickness 0.15 mm) and aluminum carbon foil (Al/C, diameter 15 mm, thickness 0.15 mm) were used in the test. The solid sulfide electrolyte Li_6PS_5Cl (LPSCl fine, NEI Corp., ~1 µm particle size) and the cathode active material (uncoated $LiNi_{0.9}Co_{0.05}Mn_{0.05}O_2$ - NMC90505, monocrystal, Chengdu B&M Science and Technology Co. Ltd) were opened in an Ar-filled glovebox and used as received, whereas the conductive additive (C65, Imerys) was vacuum dried at 100°C and then transferred to the glovebox for use. Sulfide electrolytes require special precautions and environmental conditions for storage and handling in an Ar-filled glove box with condition (H_2O < 0.1 ppm; O_2 < 0.1 ppm) [2,3]. Hazard statements specific to Li and sulfide-based electrolytes such as LPSCl include but are not limited to: flammable solid, releases flammable gas when contacted with water and toxic if swallowed. The main precautions are: handle under inert gas (typically argon) or dry atmosphere (e.g. dry room); protect from moisture - this also applies to NMC90505 as it is very sensitive to moisture; avoid inhalation of dust, vapors, gas, mist, vapor and/or spray; and wear protective gloves, clothing, eye and face protection, i.e. guidelines for handling sulfide materials and waste treatment are given in [2].

Preparation of electrolyte dry-pellets: The method used to prepare the dry-pellets with lithium metal and current collectors are described in [2]. CC|Li and Li|Li symmetric cells were assembled using densified electrolyte pellets (Figure S2 (Supplementary Information)). These were prepared as follows: around 100 mg of the Li_6PS_5Cl powder was used and placed in a 10 mm hardened steel die set (Across International, W18Cr4V hardened carbon tool steel). Around 50 MPa were applied for a couple of seconds and then the pressure was increased to 360 MPa for 2 min as shown in Figure S3 (Supplementary Information). Dense pellets with densities in the range of 1.6 to 1.55 g cm^{-3} could be thus obtained, in good agreement with literature values and the crystallographic density of 1.64 g cm^3 [4].

Preparation of bi-layer dry pellets: In the case of preparation of bi-layer pellets: Preparation of the composite was conducted in an Ar-filled glovebox (H_2O < 0.1 ppm; O_2 < 0.1 ppm) unless

otherwise stated. Composite cathodes were prepared by weighing the components, i.e., active material (AM, NCM90505), halide solid electrolyte (SE, Li_6PS_5Cl), and conductive additive (CA, carbon black, Super C65, Imerys), and manually mixing them with an agate mortar and pestle until a homogeneous mixture was obtained. After pressing at 50 MPa - cathode composite 57:40:3 weight percentage (loading of 1 mAh cm^{-2} and approximately 8–9 mg cm^{-2}) is placed on the solid electrolyte surface pellets (same method of preparation of electrolyte dry pellets as described above) from one side and compacted at a pressure of 300 MPa is maintained for 5 min (Figure S10, Supplementary Information). The lithium metal anode was then put on the opposite side of the solid electrolyte pellet and was applied a pressure of 10 MPa during the electrochemical measurements in the potential range of 2.7-4.3 V (vs. Li/Li^+) at the current density of 50 µA cm^{-2} (C/20) (Figure S11, Supplementary Information).

Ionic conductivity: Electrochemical impedance spectroscopy (EIS) was carried out to determine the bulk ionic conductivity of the solid sulfide electrolyte in symmetrical cells with stainless steel (SS) as shown in Figure S3 (Supplementary Information). The measurement was conducted at 25ºC and fitted using an equivalent circuit model on the ZView software package. An equivalent circuit model consisting of (R_b), ($R_{gb}CPE_{gb}$), ($R_{int}CPE_{int}$), and (W_s) was constructed to fit the EIS data, where R_b, R_{gb}, R_{int} and W_s are the bulk and grain boundary resistances, the resistance of the interface between the electrode and SE, and Warburg element, respectively, whereas CPE denotes constant phase element [5,6]. The ionic conductivity (σ) of the sulfide electrolyte is calculated from Equation (1):

$\sigma = l/((R(Rb+Rgb)\times A))$, Equation (1)

where l, and A are the solid electrolyte thickness and area, respectively. The pressure inside the coin cell is dependent on the sample thickness and can amount to 0.2 MPa [1]. Such rather low pressures will allow estimating the cell performance under conditions relevant for the application and avoid the fabrication of too optimistic data due to unrealistic conditions during cell operation.

Lithium plating and stripping: Stripping-plating tests were performed on symmetrical CC | LPSCl | Li/CC or CC/Li | LPSCl | Li/CC cells as described in [2] and as shown in Figure S9 (Supplementary Information). The measurements were performed in chronopotentiometry cyclic mode at a constant current density of 150 $\mu A\cdot cm^{-2}$ for 1 hour for each half of the cycle. The area of lithium metal and current collectors was 0.785 cm^2. These tests provide information on cell polarization [7], lithium deposition and dissolution kinetics [8], and dendrite propagation [9].

Storage test: Storage test of CC | LPSCl | CC samples in coin cell type configuration was performed via Electrochemical impedance spectroscopy (EIS) conducted with a Gamry Interface 1010 E from 2 MHz to 10 Hz at 10 mV amplitude in PEIS mode for 30 days.

Physicochemical analysis: The surface morphology of the samples was examined using field emission scanning electron microscopy (FE-SEM, ZEISS Supra 40) combined with energy-dispersive X-ray spectroscopy (EDS). The obtained SE pellet samples before and after contact with current collectors were determined by X-ray diffraction (XRD) analysis using a PANalytical X’Pert Pro

diffractometer in Bragg-Brentano geometry with Cu Kα radiation (45 kV, 40 mA) in a 2θ range of 5–80° at a scan rate of 0.03° s^{-1}. The elemental composition of the LPSCl powder and surface current collectors were determined using wavelength-dispersive X-ray fluorescence (WDXRF, Zetium, Panalytical, The Netherlands). The samples were irradiated with X-ray emission provided by a Rh tube with a maximum power of 4000 W, maximum voltage of 60 kV, and maximum current of 125 mA. The research was performed with the application of a fine collimator, analyzing crystal LiF200 (0.124 Å), and a scintillation counter as a detector (SC). For all elements evaluated in this study, only the electronic transition, Kα, was considered. The XPS spectra were calibrated to the standard adventitious C 1s peak at 284.8 eV. Analysis of the XPS spectra was performed using a nonlinear Shirley-type background. Core peaks and areas were fitted using a weighted least-squares method with Lorentzian line shapes.

For taking micrographs, a scanning electron microscopy (ZEISS Supra 40) was used. Acceleration voltages of 3-5 kV were applied. All samples were prepared inside an Ar filled glovebox and transported in a sealed container to the microscope. For mounting purposes, the container was opened and the samples were exposed to air for a couple of seconds, before being evacuated within the microscope sample chamber.

Supplementary Data File 1: All raw data are available in Supplementary Data File 1 (https://doi.org/10.6084/m9.figshare.29255948.v1).

Supplementary References:

[1] A. Beutl, A. Orue, P. López-Aranguren, A.I.P. Martinez, M.H. Braga, V. Kekkonen, A. Tron, Round-robin test of all-solid-state battery with sulfide electrolyte assembly in coin-type cell configuration, Electrochem. Sci. Adv. (2024) e2400004.

[2] A. Tron, A. Orue, P. López-Aranguren, A. Beutl, Critical current density measurements of argyrodite Li_6PS_5Cl solid electrolyte at ambient pressure, J. Electrochem. Soc. 170 (2023), 100525.

[3] D.H.S. Tan, A. Banerjee, Z. Deng, E.A. Wu, H. Nguyen, J.-M. Doux, X. Wang, J.-H. Cheng, S.P. Ong, Y.S. Meng, Z. Chen, Enabling thin and flexible solid-state composite electrolytes by the scalable solution process, ACS Appl. Energy Mater. 2 (2019) 6542–6550.

[4] J.-M. Doux, Y. Yang, D.H.S. Tan, H. Nguyen, E.A. Wu, X. Wang, A. Banerjeea, Y.S. Meng, Pressure effects on sulfide electrolytes for all solid state batteries, J. Mater. Chem. A 8 (2020) 5049-5055.

[5] A.O. Mendizabal, M. Cheddadi, A. Tron, A. Beutl, P. López-Aranguren. Understanding interfaces at the positive and negative electrodes on sulfide-based solid-state batteries. ACS Appl. Energy Mater. 6 (2023) 11030-11042.

[6] S. Narayanan, U. Ulissi, J.S. Gibson, Y.A. Chart, R.S. Weatherup, M. Pasta, Effect of current density on the solid electrolyte interphase formation at the lithium|Li_6PS_5Cl interface, Nat. Commun. 13 (2022) 7237.
[7] G. Bieker, M. Winter, P. Bieker, Electrochemical in situ investigations of SEI and dendrite formation on the lithium metal anode, Phys. Chem. Chem. Phys. 17 (2015) 8670--8679.
[8] K.N. Wood, E. Kazyak, A.F. Chadwick, K.-H. Chen, J.-G. Zhang, K. Thornton, N.P. Dasgupta, Dendrites and pits: untangling the complex behavior of lithium metal anodes through operando video microscopy, ACS Cent. Sci. 2, 11 (2016) 790–801.
[9] D.K. Singh, A. Henss, B. Mogwitz, A. Gautam, J. Horn, T. Krauskopf, S. Burkhardt, J. Sann, F.H. Richter, J. Janek, Li_6PS_5Cl microstructure and influence on dendrite growth in solid-state batteries with lithium metal anode, Cell Reports Physical Science 3 (2022) 101043.

Table S1. *CR2032 coin cells, uniaxial press device and pressing die set used.*

**CR2032**		**Uniaxial press device**		**Pressing die set**	
**Supplier**	**Grade**	**Supplier**	**Model**	**Supplier**	**Material**
X2lab	SS316	Maassen	MP15	Across International	W18Cr4V hardened carbon tool steel

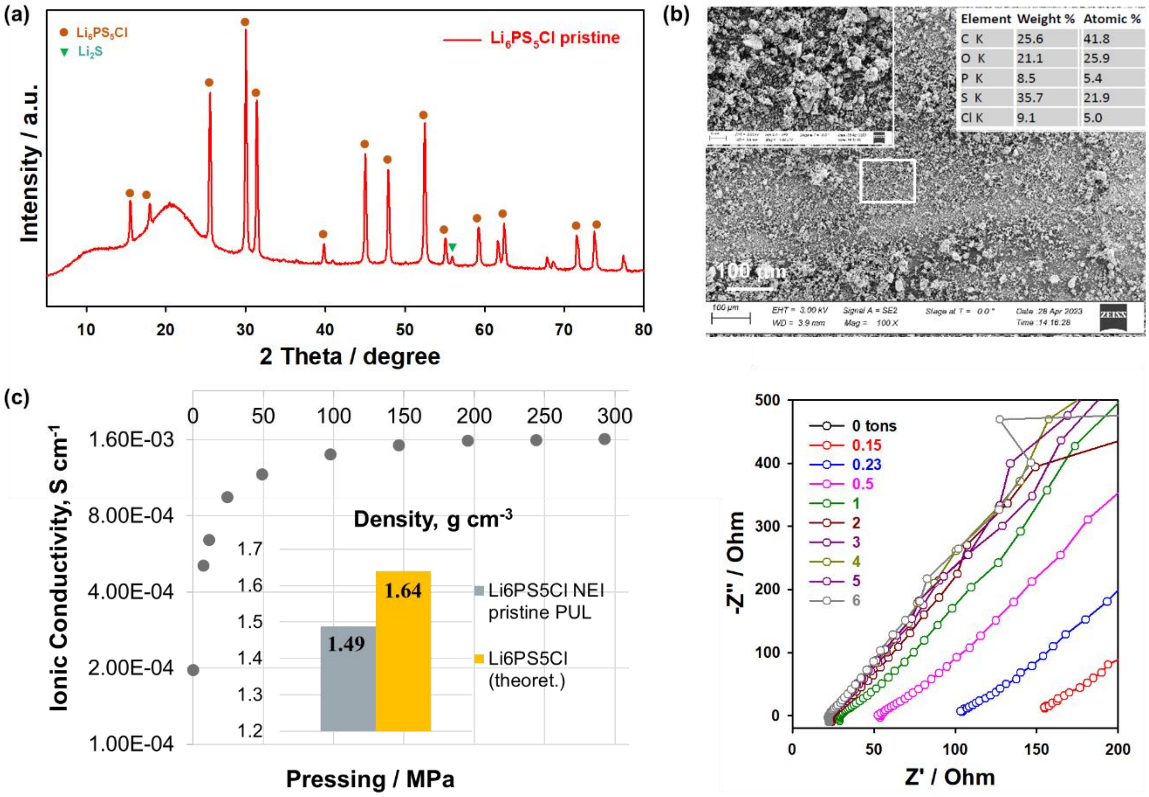

	Pressure, MPa									
Ionic Conductivity, mS cm^{-1}	0	7.32	11.22	24.39	48.77	97.55	146.32	195.09	243.87	292.65
	0.2	0.51	0.64	0.95	1.17	1.40	1.53	1.59	1.60	1.62

Figure S1. *(a) XRD pattern, (b) SEM images and EDS analysis and (c) Ionic conductivity (included Nyquist plots) and Density of Li_6PS_5Cl sulfide electrolyte before testing.*

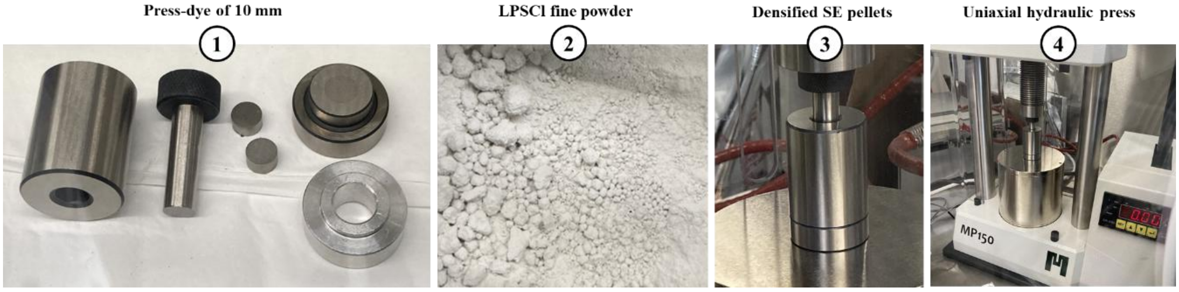

Figure S2. *Uniaxial hydraulic press and press-dye of 10 mm and prepared LPSCl pellet with a diameter of 10 mm and a thickness of 0.8 mm.*

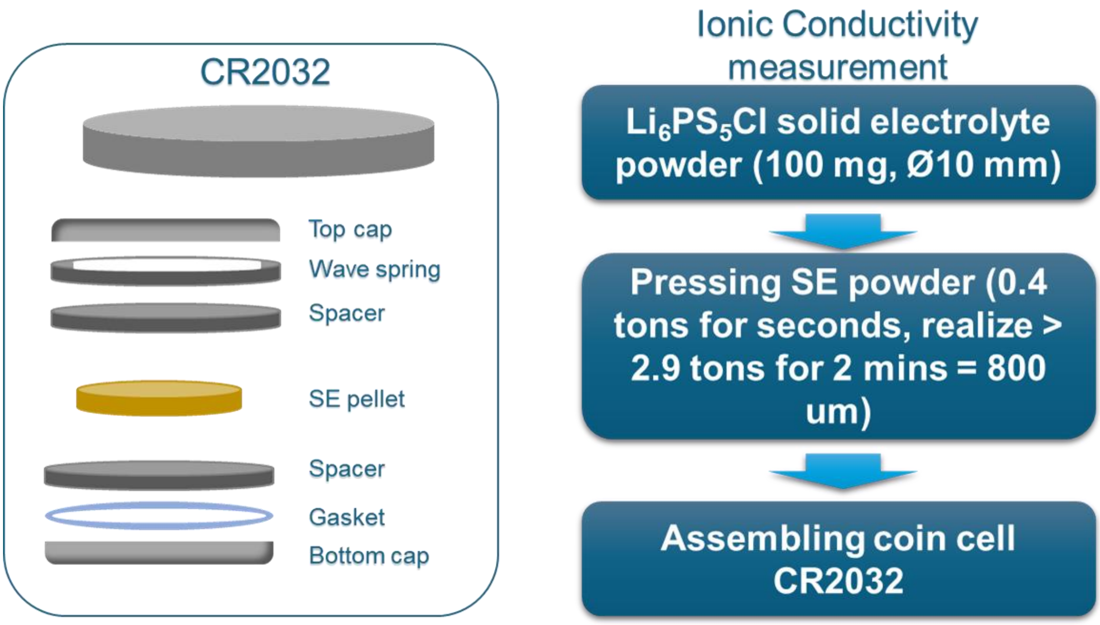

Figure S3. *Schematic illustration of assembling SSB cell of CR2032 for ionic conductivity measurement with SS current collector.*

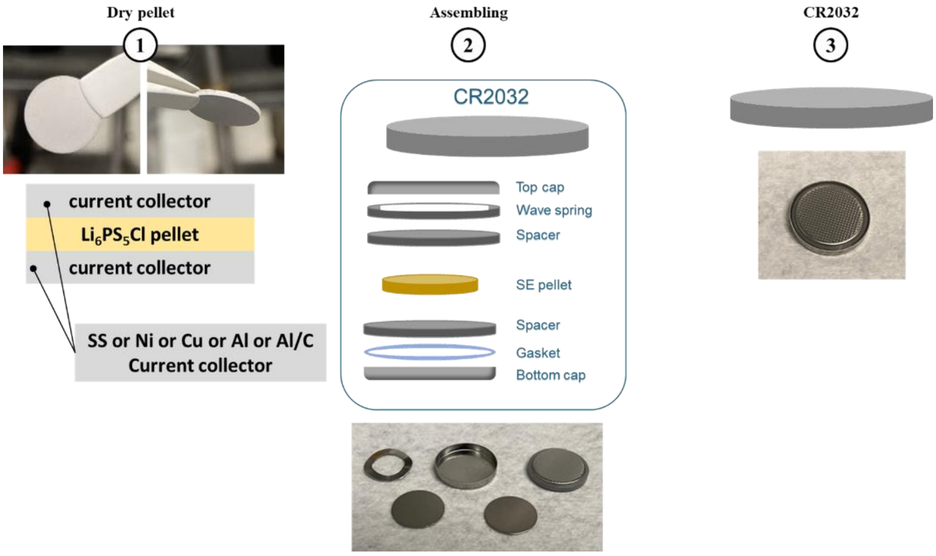

Figure S4. *Assembling steps of CC|Li and Li|Li symmetric cells using densified LPSCl electrolyte pellets with different current collectors and lithium metal anode.*

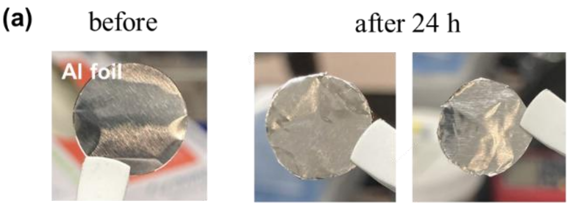

(b) after 2 months contacted with LPSCl

XRF data<br>Parameter		Aluminum Corroded
**P**	%	0.11
**S**	%	0.30
**Cl**	%	0.03
**Al**	%	99.56
SUMME	%	100.0

Figure S5. *Photos of Al current collector of (a) before and after contact with LPSCl electrolyte for 24 h, and (b) XRF analysis after 2 months.*

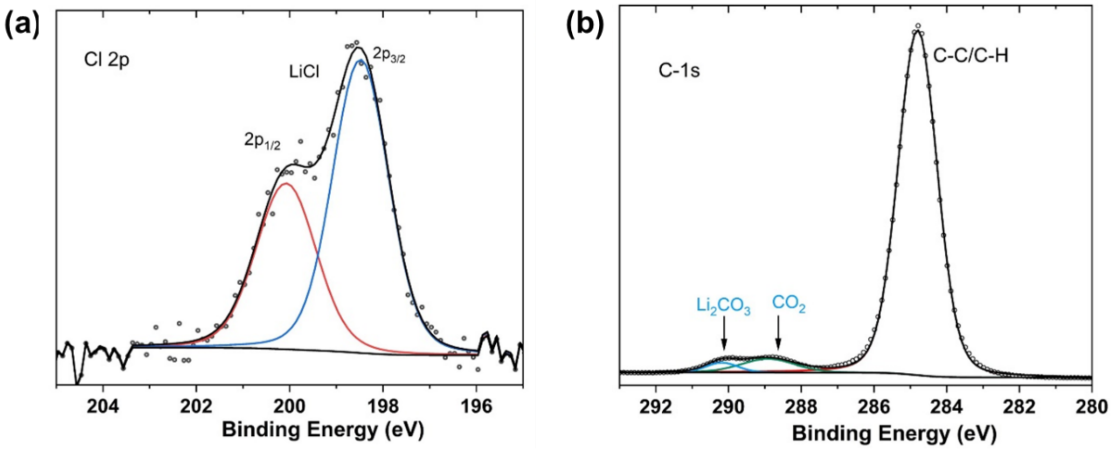

Figure S6. *The high-resolution deconvoluted XPS spectra of (a) Cl 2p for SS, and (b) for C 1s for Li current collectors.*

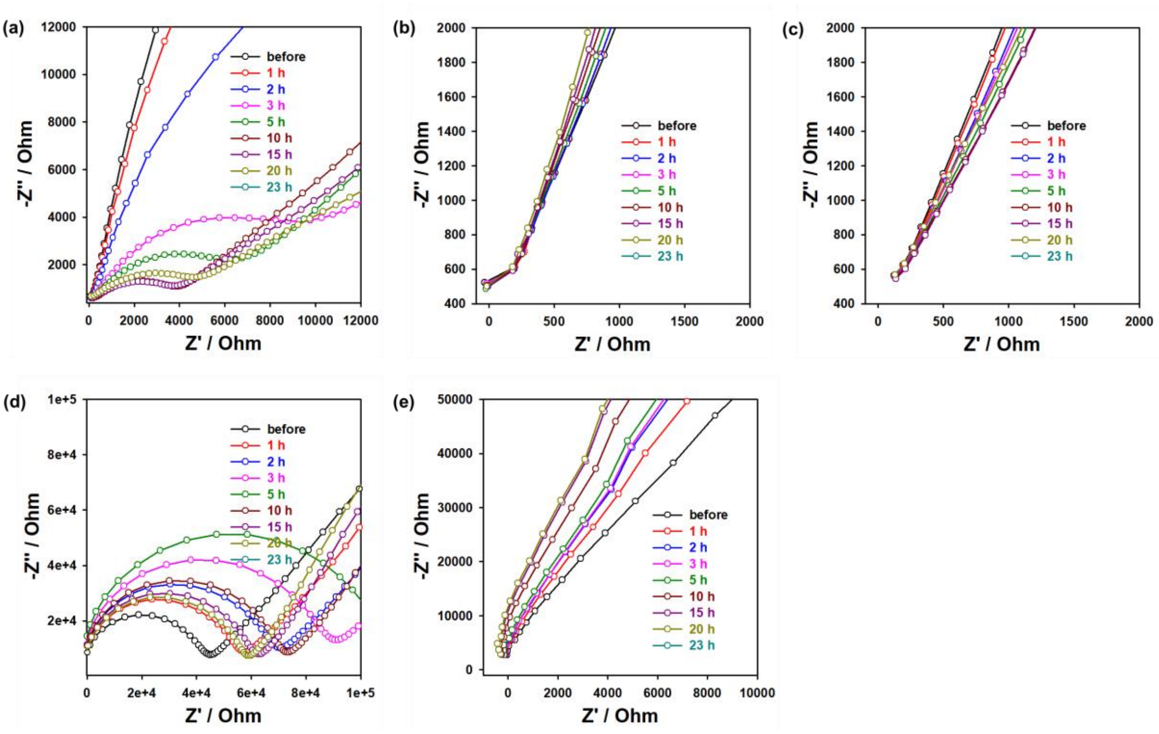

Figure S7. *Nyquist plots of current collector of (a) Cu, (b) SS, (c) Ni, (d) Al, and (e) Al/C before and after contact with LPSCl electrolyte for 24 h from* Figure 4.

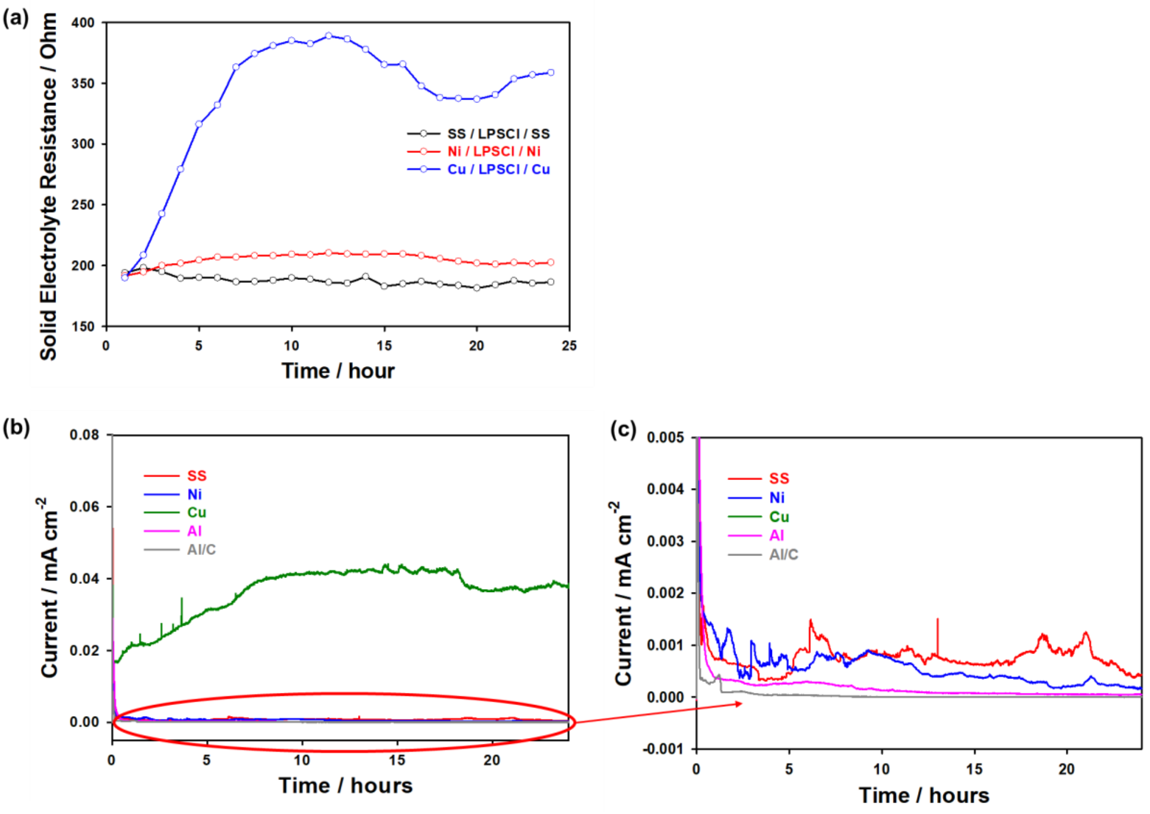

Figure S8. *(a) Resistance obtained from Nyquist plots (*Figure 4*) of current collectors of Cu, SS, Ni, Al, and Al/C before and after direct contact with LPSCl electrolyte for 24 h, and (b and c) Time and current (i–t) curves of current collectors after scratching at 4.3 V polarized in LPSCl electrolyte.*

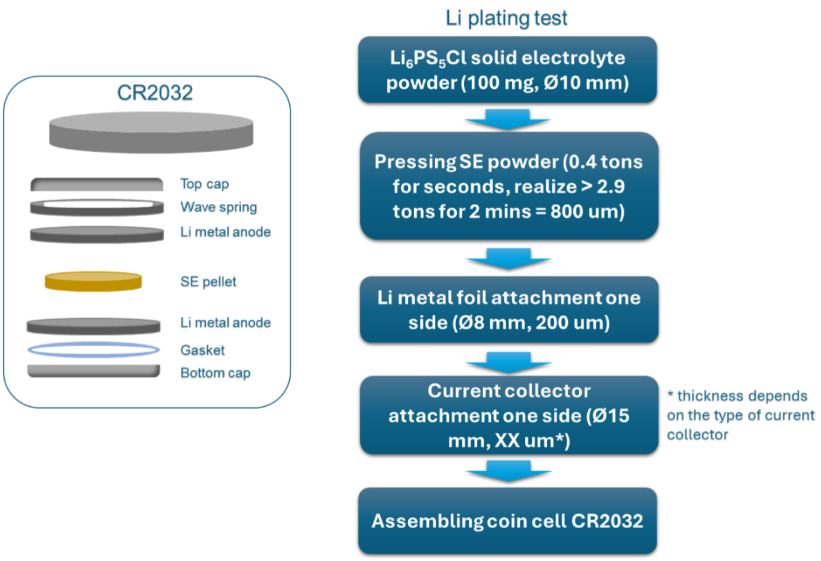

Figure S9. *Schematic illustration of assembling SSB cell of CR2032 for Li plating/stripping test and Storage test with different current collectors of Cu, SS, Ni, Al and Al/C.*

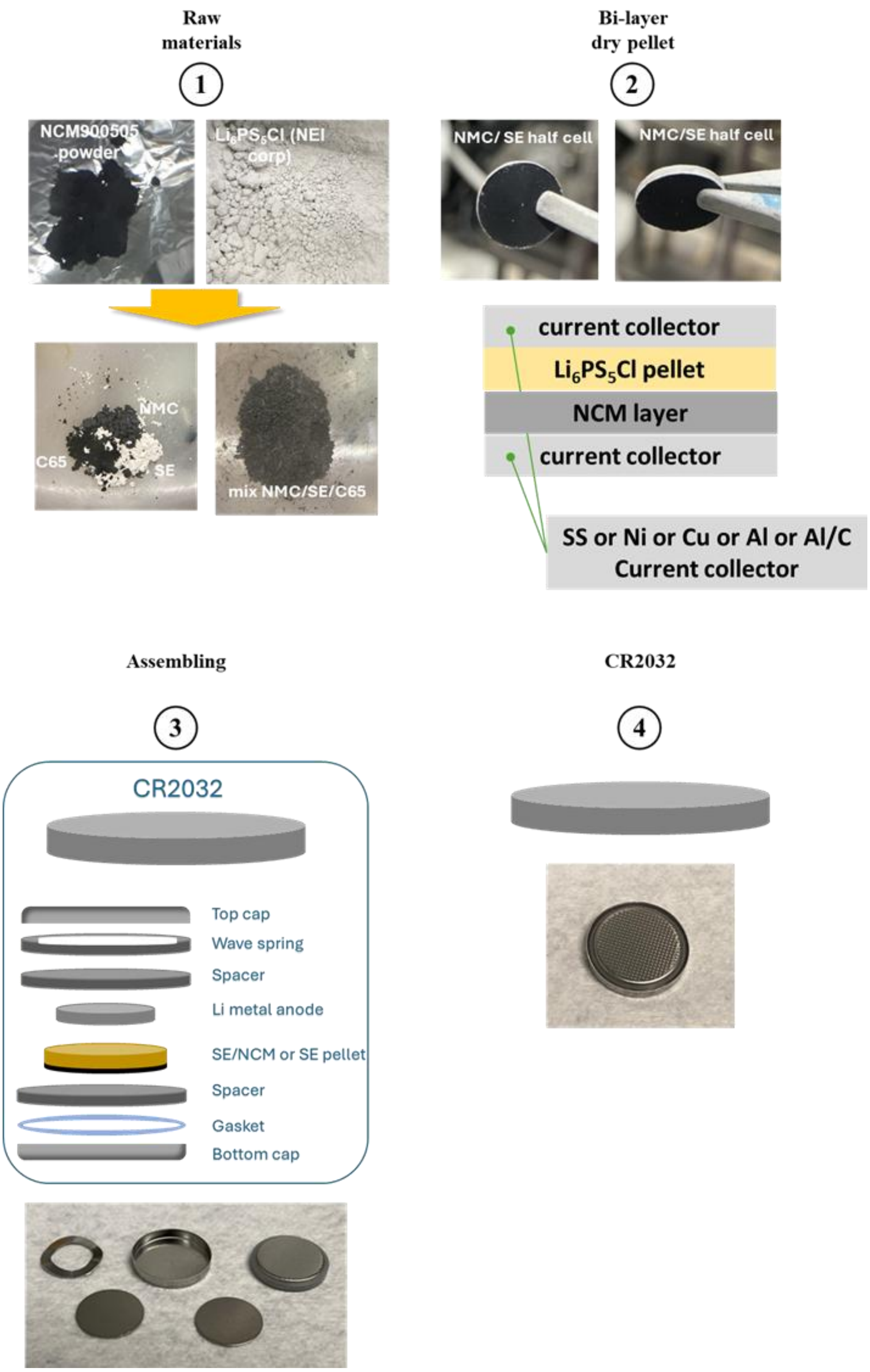

Figure S10. *Assembling steps of Li|SE|NCM cells with different current collectors.*

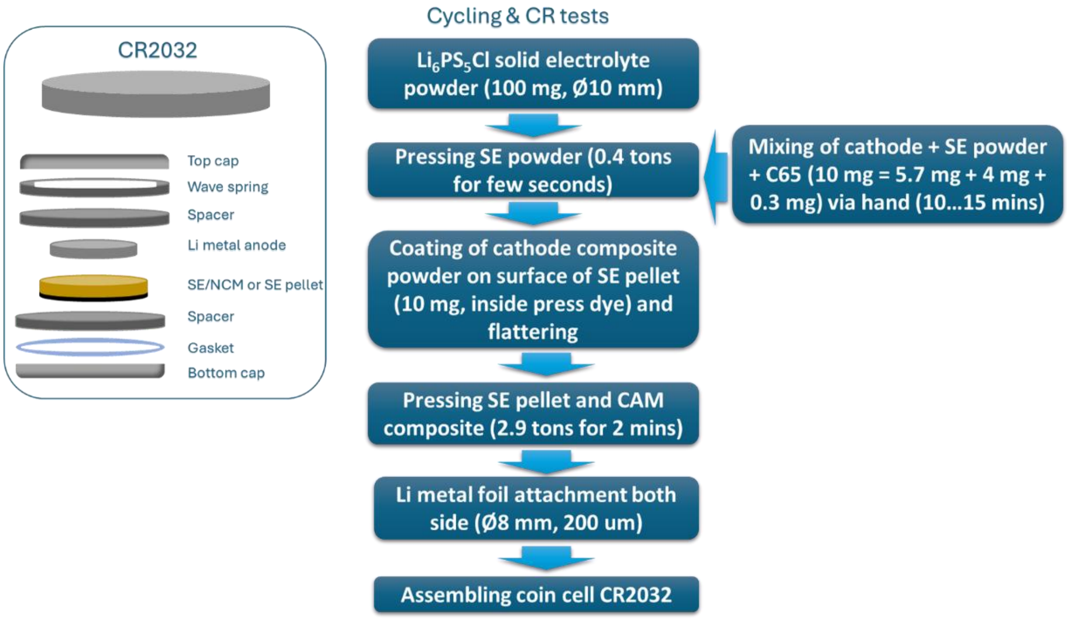

Figure S11. *Schematic illustration of assembling SSB cell of CR2032 for cycling test of a full cell of Li|SE|NCM with different current collectors of Cu, SS, Ni, Al and Al/C.*

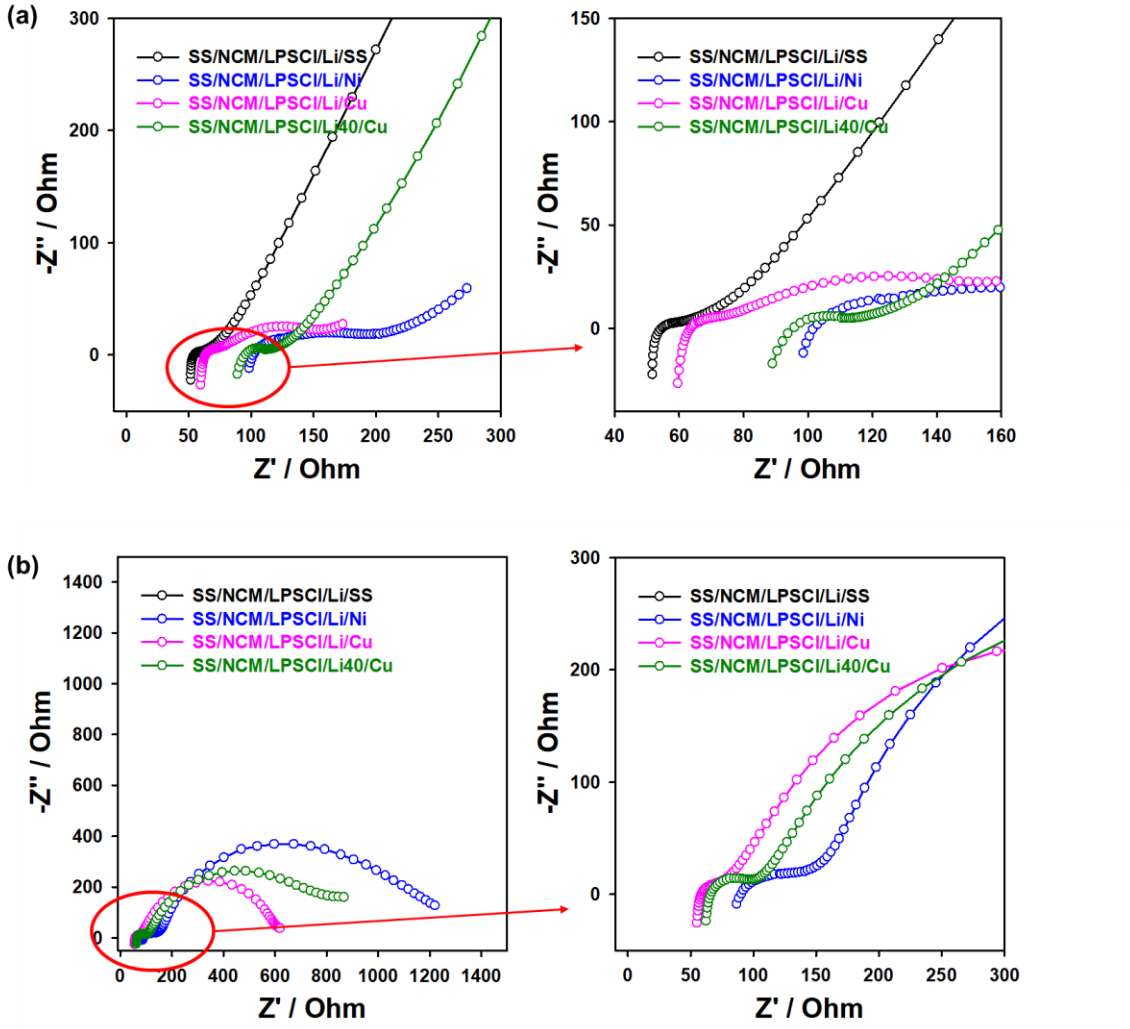

Figure S12. *Nyquist plots of (a) before and (b) after of NCM90505 | LPSCl | Li/SS cell, NCM90505 | LPSCl | Li/Ni cell, NCM90505 | LPSCl | Li/Cu cell, and NCM90505 | LPSCl | Li40/Cu (thickness of lithium on Cu foil is 40 μm) cell obtained from* Figure 6 *in a potential range of 2.7 and 4.3 V at room temperature at C/20 into coin cell format CR2032.*
