# Supplementary material for: Probing the chemical stability between current collectors and argyrodite Li6PS5Cl sulfide electrolyte
Source: Commun Chem. 2025 Jul 24;8:212. doi: 10.1038/s42004-025-01609-9 (PMC12289912; doi:10.1038/s42004-025-01609-9)
Supplement: Supplementary file 3 — Description of Additional Supplementary Files [file 42004_2025_1609_MOESM3_ESM.pdf]

Description of Additional Supplementary Files

File name: Supplementary Data 1

Description: Raw data for all experimental components, including physicochemical and electrochemical analyses, are provided as source data for the main figures in both the primary publication and the supplementary information.
